# Whole exome sequencing and proteomics-based investigation of the pathogenesis of coronary artery disease with diffuse long lesion

**DOI:** 10.1186/s13019-024-02760-5

**Published:** 2024-05-07

**Authors:** Ce Chao, Yongxiang Qian, Hao Lv, Kun Mei, Min Wang, Yang Liu, Bin Wang, Dongmei Di

**Affiliations:** Department of Cardiothoracic Surgery, The Third Affiliated Hospital of Soochow University, No.185, Juqian Street, Tianning District, Changzhou, 213003 Jiangsu Province China

**Keywords:** Whole exome sequencing, Proteomics, Coronary artery disease, Diffuse long lesion, Gene variant, Pathogenesis

## Abstract

**Objectives:**

The long-term prognosis of patients with coronary artery disease (CAD) with diffuse long lesion underwent coronary artery bypass graft (CABG) or percutaneous coronary intervention (PCI) remains worse. Here, we aimed to identify distinctive genes involved and offer novel insights into the pathogenesis of diffuse long lesion.

**Materials and methods:**

Whole exome sequencing was performed on peripheral blood samples from 20 CAD patients with diffuse long lesion (CAD-DLL) and from 10 controls with focal lesion (CAD-FL) through a uniform pipeline. Proteomics analysis was conducted on the serum samples from 10 CAD-DLL patients and from 10 controls with CAD-FL by mass spectrometry. Bioinformatics analysis was performed to elucidate the involved genes, including functional annotation and protein–protein interaction analysis.

**Results:**

A total of 742 shared variant genes were found in CAD-DLL patients but not in controls. Of these, 46 genes were identified as high-frequency variant genes (≥ 4/20) distinctive genes. According to the consensus variant site, 148 shared variant sites were found in the CAD-DLL group. The lysosome and cellular senescence-related pathway may be the most significant pathway in diffuse long lesion. Following the DNA-protein combined analysis, eight genes were screened whose expression levels were altered at both DNA and protein levels. Among these genes, the *MAN2A2* gene, the only one that was highly expressed at the protein level, was associated with metabolic and immune-inflammatory dysregulation.

**Conclusions:**

Compared to individuals with CAD-FL, patients with CAD-DLL show additional variants. These findings contribute to the understanding of the mechanism of CAD-DLL and provide potential targets for the diagnosis and treatment of CAD-DLL.

**Supplementary Information:**

The online version contains supplementary material available at 10.1186/s13019-024-02760-5.

## Introduction

Coronary artery disease (CAD) is partially a genetic disease. Family and twin studies have shown that there is 40 ∼ 60% heritability for patients with CAD [1]. With regard to the pathological mechanisms, CAD mainly results from atherosclerosis caused by cholesterol accumulation. With the progression of CAD, myocardial contractility and cardiac function gradually decrease, eventually leading to heart failure [2]. Presently, coronary artery bypass grafting (CABG) and percutaneous coronary intervention (PCI) are the primary treatment approaches for CAD [3]. CAD with diffuse long lesion is a complex disorder with poor prognosis [4]. Although some patients with diffuse long lesion undergo PCI treatment in the acute stage, more patients are required to undergo reoperation [5]. A majority of patients who have received CABG are in the stable phase. However, the presence of a long lesion may easily lead to incomplete revascularization and early graft occlusion due to poor distal flow. Thus, it is challenging for cardiovascular surgeons to improve the prognosis and reduce the risk of vein graft restenosis in CAD patients with diffuse long lesion (CAD-DLL).

Next-generation sequencing (NGS) can identify single nucleotide polymorphisms (SNPs) in the entire genome and can enable to elucidate the diagnosis and pathogenesis of diseases from the aspect of heredity [6]. Whole exome sequencing (WES), a routine sequencing technique, is widely used to diagnose genetic diseases [7]. By screening genetic variations of the genes involved in various diseases and comparing them with the normal gene database, researchers can provide additional molecular diagnosis of the diseases. For example, Ji et al. identified 4 gene variants and a CYP2W1 deletion in patients with vascular intestinal obstruction by using WES. The pathological mechanism of vascular intestinal obstruction was explained for the first time on the basis of genetic variants [8]. For CAD, previous studies have conducted exome sequencing to identify causative variants for cardiovascular risk in early-onset CAD patients with familial metabolic syndrome [9]. Some researchers have used whole exome sequencing (WES) to screen genetic variants in patients with CAD, myocardial infarction, and familial metabolic syndrome to develop methods for preventing and treating CAD [10, 11]. However, few studies have focused on CAD patients with diffuse long lesion. It is hypothesized that these patients carry some congenital factors that lead to the formation of diffuse long lesion more easily.

In the present study, we used WES and proteomics-based approaches to identify specific variant genes in CAD patients with diffuse long lesion. For this purpose, peripheral blood samples were collected from 20 CAD patients with diffuse long lesion and 10 controls with focal lesions. The purpose of this study was to improve the prognosis of patients with diffuse long lesion and prevent vein graft restenosis.

## Materials and methods

### Patients and peripheral blood samples

This study included 30 unrelated patients with coronary heart disease (CHD) from the Third Affiliated Hospital of Soochow University. Of these patients, 20 patients had complex long lesions in the coronary artery and 10 patients had only focal lesions in the coronary artery. All patients with CHD were diagnosed by coronary angiography. Peripheral blood samples were collected from these 30 patients preoperatively after hospitalization. The samples were frozen and stored in a -80℃ refrigerator for the subsequent WES and proteomics analyses. All patients had no previous history of illness. The matched clinicopathologic data were also collected. The study was approved by the Ethical Review Board of the Third Affiliated Hospital of Soochow University. Written informed consent was obtained from all subjects prior to sample collection.

### WES

Genomic DNA was extracted from peripheral blood samples by using standard methods. Agarose gel electrophoresis was performed to analyze DNA content, purity, and degradation. The Agilent SureSelect Human All Exon V6 Kit (Agilent Technologies, Santa Clara, CA, USA) was used for exome capture from 20 genomic DNA samples according to the manufacturer’s instructions. The captured exome was then sequenced on the Illumina Novaseq 6000 platform (Illumina Inc., San Diego, CA, USA). The resulting fastq data were entered into in-house quality control software for removing low-quality reads and then aligned with the reference human genome (hs37d5) by using the Burrows-Wheeler Aligner (bwa) [12]. Duplicate reads were marked using sambamba tools [13]. SNP/INDEL calling: single nucleotide variants (SNVs) and indels were called with samtools to generate gVCF [14]. The raw calls of SNVs and INDELs were further filtered with the following inclusion thresholds: (1) read depth > 15; (2) Root-Mean-Square mapping quality of covering reads > 30; and (3) the variant quality score > 20. The copy number variation (CNV) was detected with software CoNIFER (V0.2.2) [15]. All these variants were annotated based on ANNOtate VARiation (ANNOVAR) [16]. According to the ANNOVAR, it identified variants that are documented in specific databases, such as the 1000 Genome Project, NHLBI-ESP 6500 exomes or Exome Aggregation Consortium (ExAC) or Genome Aggregation Database (gnomAD). And it can find intergenic variants with GERP + + score < 2 or CADD > 10, or many other annotations on specific mutations.

### Rare variant filtering

Filtering of rare variants was performed as follows: (1) minor allele frequency (MAF) less than 0.01 in 1000 genomic data (1000g_all), esp6500siv2_all, and gnomAD data (gnomAD_ALL and gnomAD_EAS); (2) only SNVs occurring in exons or splice sites (splicing junction 10 bp); and (3) synonymous SNVs not relevant to the amino acid alternation predicted by dbscSNV were discarded. The small-fragment non-frameshift (< 10 bp) indels in the repeat region defined by RepeatMasker were discarded. To determine whether high-frequency variant genes were intolerant to variance, we used the probability of loss-of-function intolerance (pLI) score (https://gnomad.broadinstitute.org/) and the residual variation intolerance scores (RVIS) [17], which are both reliable measures of genic intolerance to ldDNMs [18]. Higher pLI and lower RVIS values indicate greater intolerance to functional and deleterious missense variants in a gene, respectively. According to the previous study, chromosome 9p21 and 6p24 regions were strongly associated with CAD [19]. Therefore, we screened the variants in the chromosome 9p21 and 6p24 regions.

### Proteomics

Serum samples from 20 subjects (10 CAD-DLL patients and 10 CAD-FL subjects) were analyzed by DIA quantitative proteomics analysis. The total protein was extracted and quantified by the BCA Protein Assay Kit (P0012, Beyotime). The proteins were digested with trypsin, and the resulting peptides were collected as a filtrate. Pooled peptides from all samples were fractionated by reversed-phase chromatography using the Agilent 1260 infinity II HPLC. A protein spectrum library was established using the data-dependent acquisition (DDA) method, and mass spectrometry data of each sample were obtained using the data-independent acquisition (DIA) method. Raw data of DDA were processed and analyzed by Spectronaut X (Biognosys AG, Switzerland) with default settings to generate an initial target list. Spectronaut was configured to search the database of Uniprot_HomoSapiens_20394_20210127. Carbamidomethyl (C) was specified as the fixed modification. Oxidation (M) and acetyl (Protein N-term) were specified as the variable modifications. Qvalue (false discovery rate [FDR]) cutoff on precursor and protein level was applied as 1%. For the DIA data, the quantification of FDR was set to 0.01, and the quantity MS-level was set MS2. Following Student’s t-test, differentially expressed proteins were filtered if their *p* value were > 0.05 and with 0.83 < fold change < 1.2.

### Functional annotation and protein–protein interaction (PPI) analysis

Gene ontology (GO) analysis and Kyoto Encyclopedia of Genes and Genomes (KEGG) pathway analysis were performed to explore the biological process, molecular function, cellular component, and related pathways of the high-frequency variant genes according to the DAVID website (https://david.ncifcrf.gov/home.jsp). PPI analysis was then performed using the GeneMania online analysis tool (https://genemania.org).

### Statistical analysis

Fisher’s exact test for categorical data was used to compare clinical characteristics between the two groups. The Shapiro-Wilk test was used to investigate the normality assumption for continuous variables. If the normality assumption was true, Student’s t-test was used; otherwise, the Mann–Whitney U test was used. All statistical analyses were performed using SPSS 23.0 (SPSS Inc., Chicago, IL, USA) and R software (version 4.1.2).

## Results

### Clinical characteristic of the study patients

The clinical characteristic of the study patients in the two groups are summarized in Table 1. Compared to the control subjects, the CAD-DLL group showed significant differences in terms of age and body mass index (BMI) (*P* = 0.031 and 0.028 for age and BMI, respectively). However, no differences were observed between the two groups in terms of underlying chronic diseases and plasma lipid and lipoprotein markers, such as total cholesterol (TC), triglyceride (TG), high-density lipoprotein (HDL)-cholesterol, and low-density lipoprotein (LDL)-cholesterol levels.

**Table 1:** Baseline characteristics of coronary artery disease patients with diffuse long lesion and control subjects

Characteristics	Patients with CAD-DLL (*N* = 20)	Patients with CAD-FL (*N* = 10)	*P*-value
Age (years, mean ± SD)	66.45 (8.51)	59.60 (8.72)	**0.031** ^**a**^
Male (%)	10 (50.0)	8 (80.0)	0.235
Hypertension (%)	15 (75.0)	6 (60.0)	0.431
Diabetes mellitus (%)	11 (55.0)	3 (30.0)	0.260
Myocardial Infarction (%)	6 (30.0)	3 (30.0)	1.000
BMI (kg/m^2^, mean ± SD)	24.08 (2.84)	26.69 (3.07)	**0.028** ^**b**^
Total cholesterol (nmol/L, mean ± SD)	4.42 (1.27)	4.75 (0.67)	0.443^b^
Triglycerides (nmol/L, mean ± SD)	2.69 (3.19)	2.62 (1.24)	0.262^a^
HDL-cholesterol (nmol/L, mean ± SD)	0.94 (0.17)	0.97 (0.26)	0.730^b^
LDL-cholesterol (nmol/L, mean ± SD)	2.44 (0.93)	2.76 (0.62)	0.276^b^
Apolipoprotein A1 (g/L, mean ± SD)	1.12 (0.15)	1.21 (0.21)	0.193^b^
Apolipoprotein B (g/L, mean ± SD)	0.85 (0.32)	1.02 (0.20)	0.130^b^
Glucose (nmol/L, mean ± SD)	6.53 (2.70)	5.84 (1.53)	0.860^a^

Abbreviation: CAD, coronary artery disease; BMI, body mass index; HDL, high-density lipoprotein; LDL, low-density lipoprotein. a: Mann–Whitney test; b: Student’s t test

### Screening of shared variant genes

**Fig. 1:**
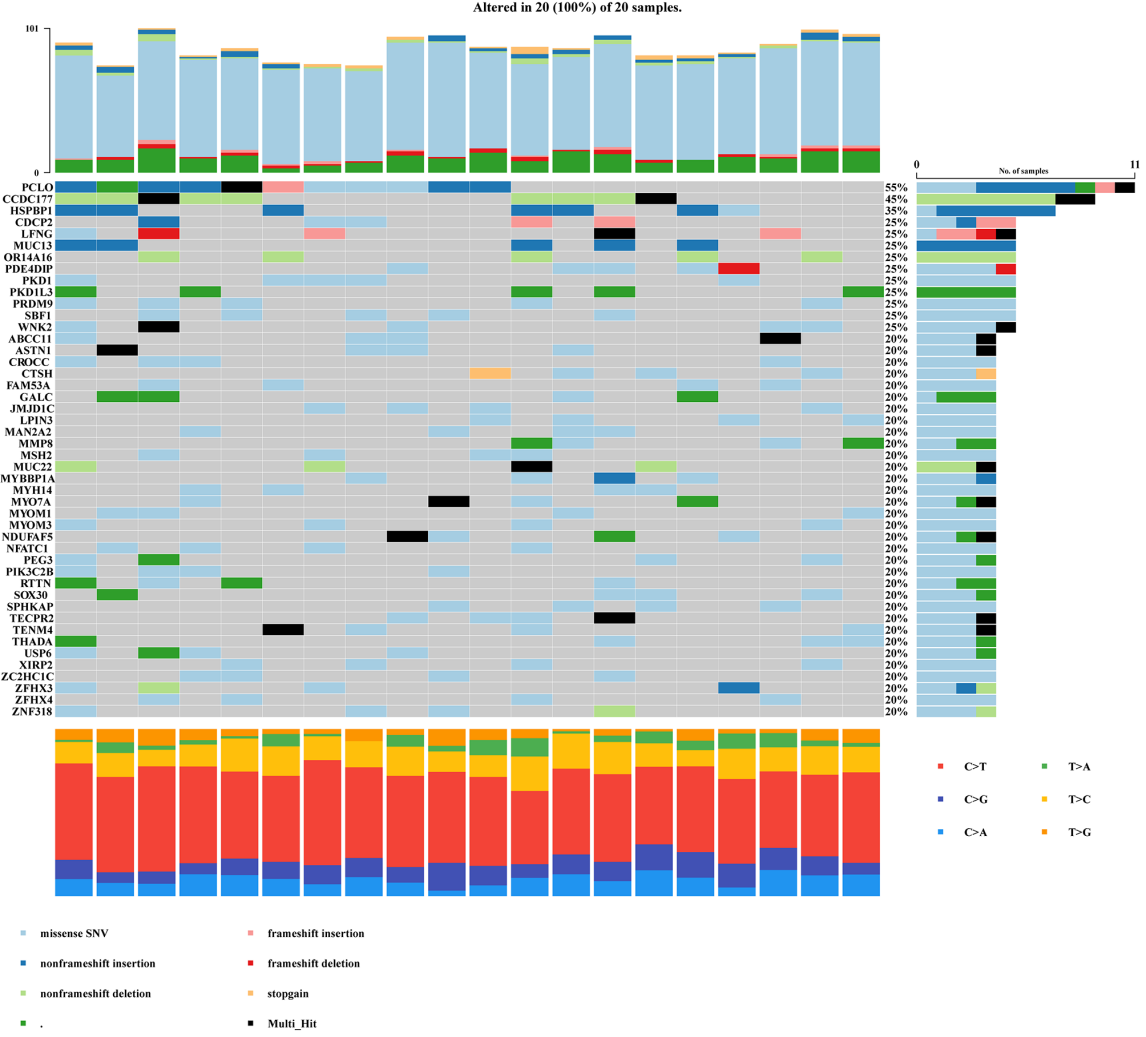
Mutational landscape of the high frequency variant genes in 20 patients with CAD-DLL. Abbreviation: CAD-DLL, coronary artery disease with diffuse long lesion

MAF < 0.01 was considered as the main variant of the disease. On the basis of this value, we screened shared variant genes in patients with CAD-DLL. A total of 742 shared variant genes were detected in patients with CAD-DLL, but no such gene variants were detected in controls with CAD-FL (Figure S1). In this context, high-frequency variant implied a variant existing in at least 4/20 patients with CAD-DLL. As shown in Fig. 1, there were 46 high-frequency variant genes in the study group. The top 3 variant genes included *PCLO*, *CCDC177*, and *HSPBP1*. And, the pLI and RVIS for the 46 high-frequency variant genes were shown in Table S2. The SBF1 and ZFHX3 with higher pLI and lower RVIS were considered as highly intolerant genes. *FUCA2, GRM1, SASH1, DMRTA1, IFT74*, and *TEK* were site in chromosome 9p21 and 6p24 regions, which are locus particularly strongly associated with CAD (Table S1).

### Identification of high-frequency variant sites

Because the same gene contains many different variant sites, it was important to identify significant and high-frequency variant sites for the subsequent intervention and treatment of patients with CAD. In this context, a high-frequency variant site implied a variant existing in at least 4/20 patients with CAD-DLL. As shown in Table 2, *PCLO, CCDC177, OR14A16*, and *PRDM9* were the high-frequency variant genes in 20 patients with CAD-DLL. The American College of Medical Genetics & Genomics (ACMG) classifications were PM1 and PM4, which suggested moderate evidence of disease.

**Table 2:** A list of the high-frequency variant genes that vary in the same variant site

Gene name	Gene annotation	Site variant frequency	Homozygous/ Heterozygous variants	ACMG	HGVS	Variant site	ConsDetail	Nucleotide alteration
CCDC177	Coiled-coil domain containing 177	9/20	0/9	PM4	NM_001271507 c.541_546delGGCCGA (p.181_182del)	14q24.1 exon2	Nonframeshift deletion	GGGCCGA > G
PCLO	Piccolo presynaptic cytomatrix protein	5/20	5/0	PM4	NM_014510 c.8776_8777insCTG (p.D2926delinsAD)	7q21.11 exon5	Nonframeshift insertion	T > TCAG
OR14A16	Olfactory receptor family 14 subfamily a member 16	4/20	4/0	PM4	NM_001001966 c.489_491delAAG (p.163_164del)	1q44 exon1	Missense	TAAG > T
PRDM9	Pr domain-containing protein 9	4/20	0/4	PM1	NM_001310214 c.2440 A > C (p.S814R)	5p14.2 exon11	Nonframeshift deletion	A > C

Abbreviation: ACMG, American College of Medical Genetics & Genomics; HGVS, Human Genome Variation Society

### GO and KEGG pathway enrichment analyses of the high-frequency variant genes

**Fig. 2:**
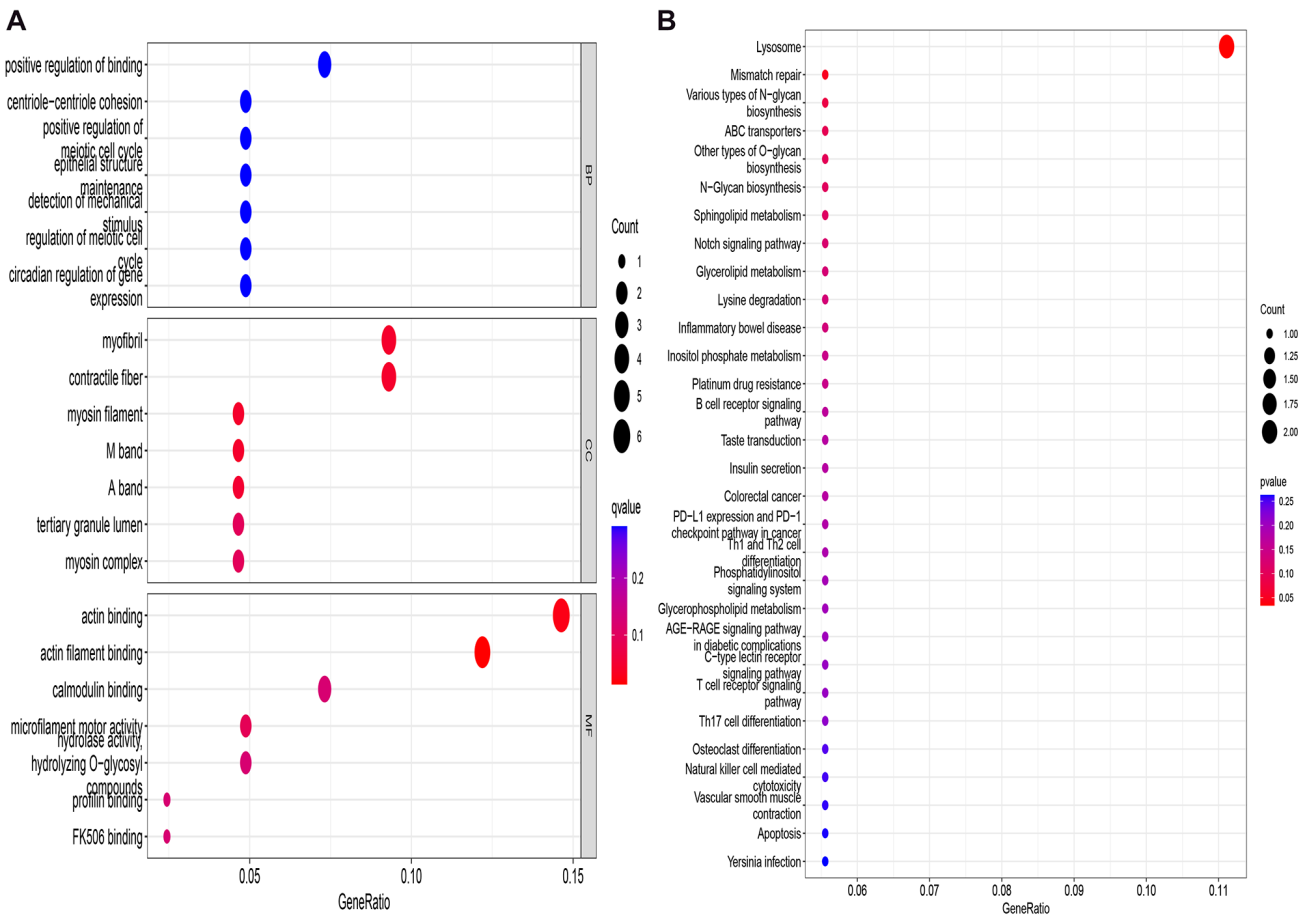
GO and KEGG enrichment analysis of 46 high frequency variant genes. (A) Biological Process (BP); (B) Cellular Component (CC); (C) Molecular Function (MF); (D) Kyoto Encyclopedia of Genes and Genomes (KEGG) enrichment analysis

To investigate the biological functions of the high-frequency variant genes, GO analysis was performed to classify the genes. According to GO analysis, these genes were enriched in biological process (BP), cellular component (CC), and molecular function (MF). As shown in Fig. 2A, the most enriched term in BP, CC, and MF were positive regulation of binding, myofibril, and actin binding, respectively. KEGG enrichment analysis was used to investigate the related pathways of the high-frequency variant genes. The results showed that only one pathway was identified as the significant pathway (*P* < 0.05). The lysosome-related pathway was found to be related to CAD-DLL (Fig. 2B).

### PPI analysis

**Fig. 3:**
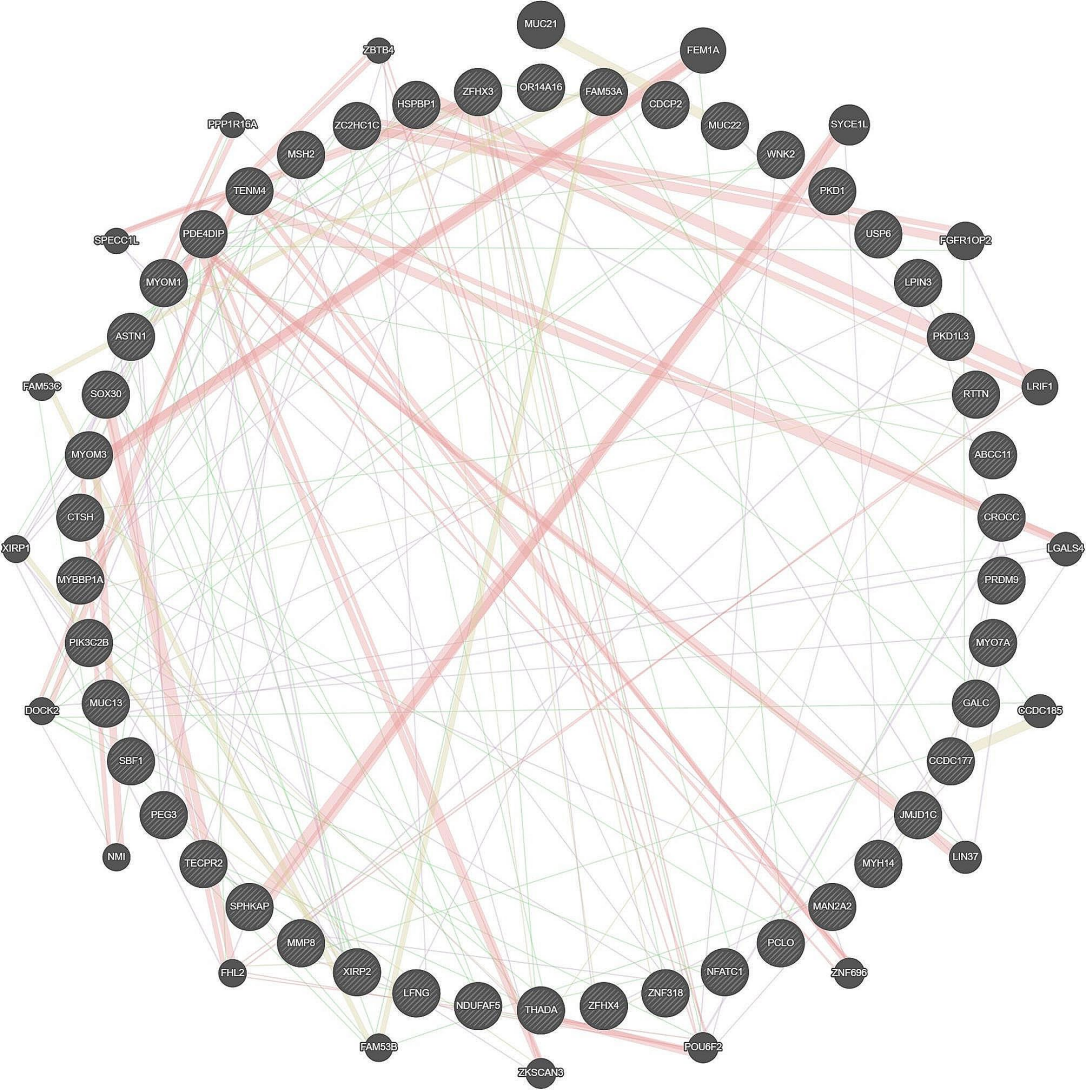
The Protein-Protein Interaction Networks (PPI) of 46 high frequency variant genes. Abbreviation: CAD-DLL, coronary artery disease with diffuse long lesion; CAD-FL, coronary artery disease with focal lesion

The protein functional interaction network analysis was conducted to further investigate the potential interactions among the high-frequency variant genes by using the online software GeneMania. Figure 3 shows the PPI network. The 46 high-frequency variant genes showed direct or indirect relationship with each other.

### DNA and protein combined analysis results

**Fig. 4:**
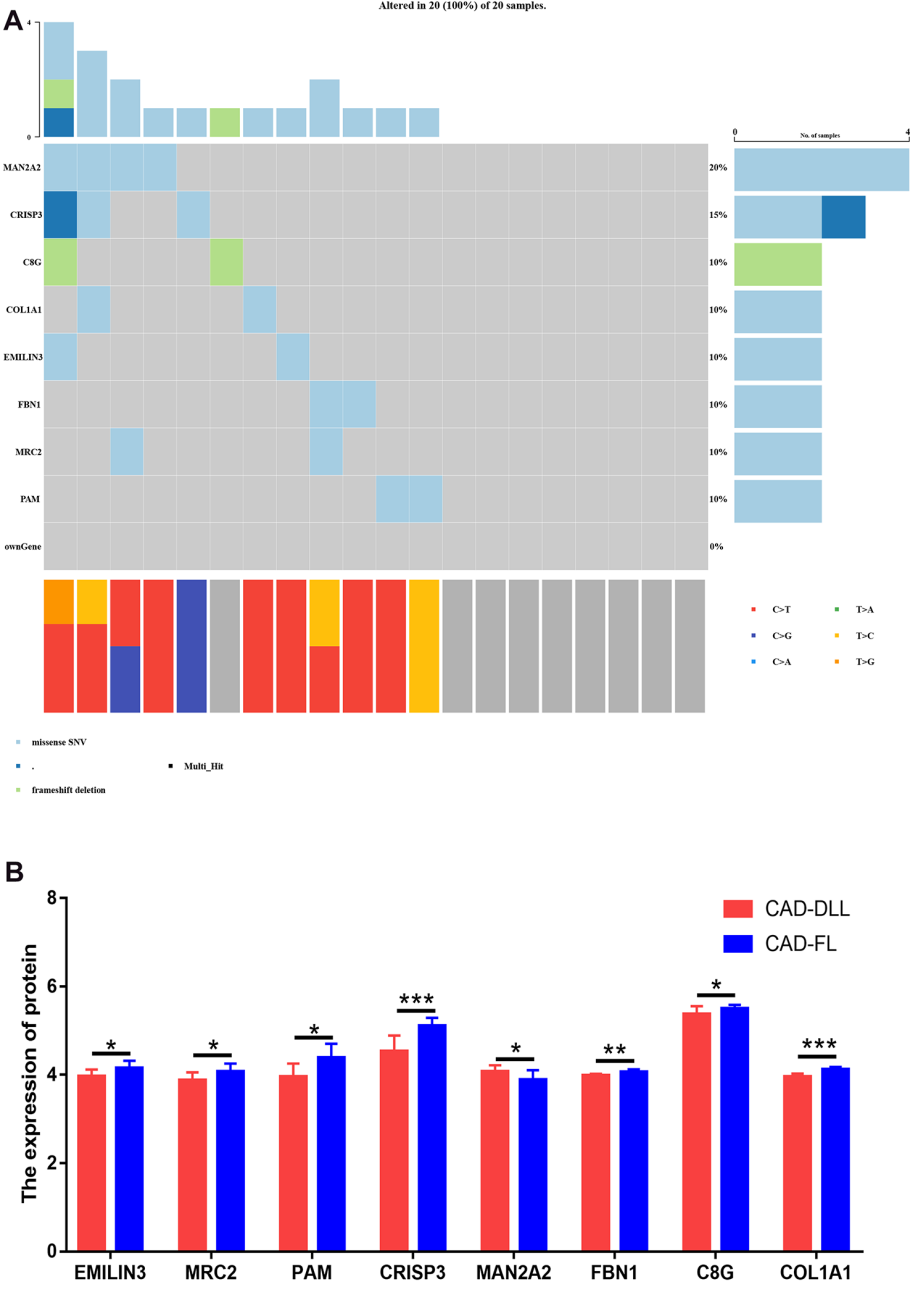
DNA-protein combined analysis screened 8 genes that synchronized changes in DNA and protein levels. (A) mutational landscape of 8 genes; (B) the protein expression level of 8 genes in patients with CAD-DLL compared with patients with CAD-FL. *P < 0.05; **P < 0.01; ***P < 0.001. Abbreviation: CAD-DLL, coronary artery disease with diffuse long lesion; CAD-FL, coronary artery disease with focal lesion

The proteomics analysis revealed 160 differentially expressed proteins. Through the intersection with shared variant genes, eight shared variant genes were identified to have significantly differential expression. Figure 4 shows the expression level of proteins and variant genes. Among these genes, Golgi alpha-mannosidase IIX (MAN2A2) was the only gene to be overexpressed in patients with CAD-DLL and had a high variant frequency. The protein level of genes *COL1A1*, *C8G*, *CRISP3*, *EMILIN3*, *FBN1*, *MRC2*, and *PAM* were downregulated in patients with CAD-DLL as compared to that in subjects with CAD-FL. The detailed variant information was shown in Table S3.

## Discussion

The present study is the first to investigate the pathogenesis of CAD-DLL from the genetic standpoint. In our study, peripheral blood samples were collected from 30 sporadic cases (20 patients with CAD-DLL and 10 patients with CAD-FL), and WES and proteomics analysis were conducted. The shared variant genes and sites were screened to investigate the potential characteristic genes of patients with CAD-DLL as compared to patients with CAD-FL. Eight variant genes with significantly different expression were identified as the potential participants in the pathogenesis of CAD according to WES and proteomics.

In the traditional view, CAD-DLL evolved from CAD-FL. In this study, we found that there were not significant differences in lipid metabolism and cholesterol metabolism between two groups. Moreover, the BMI was lower in patients with CAD-DLL. Therefore, we wanted to explore the differences in gene variants in CAD-DLL from CAD-FL. Compared to patients with CAD-FL, patients with CAD-DLL carried 742 shared variant genes. Among these genes, 46 shared variant genes were identified as high-frequency variant genes. The genes *PCLO* and *CCDC177* were the top 2 variant genes based on the variant frequency and site. The gene *PCLO* encodes a presynaptic cytomatrix protein and carried variant in 11 patients with CAD-DLL. However, to date, no studies have proved the relationship between the *PCLO* gene and the coronary artery. An increasing number of studies have shown that the *PCLO* gene variant is related to major depressive disorder and is associated with gray matter volume reduction in the left temporal lobe [20, 21]. Moreover, the rare missense variants Ser1535Leu and His5142Arg of PCLO were found in patients with bipolar disorder [22]. Patients with depressive disorder are more likely to develop CAD than the general population [23, 24]. The progression of CAD may aggravate through the crosstalk of the depression disorder with the *PCLO* gene variant and CAD. Nine patients carried a nonframeshift deletion at the same site in the *CCDC177* gene. This variant occurred in chromosome 14q24.1, which is a tumor mutation-prone region [25]. CCDC177 has been proven to have a significant prognostic value in patients with lung squamous cell carcinoma [26]. The relationship between CCDC177 and CAD is not clear according to the existing literature. Nevertheless, we speculated that the *CCDC177* gene might play a role in the pathogenesis of CAD-DLL. The PRDM9 gene, one of the most mutated genes with pan-cancer in the PR/SET domain gene family (PRDM), is associated with meiotic recombination [27–29]. And, a case reported that fetuses with a 5p14.3-p14.1 deletion may present congenital heart disease on prenatal ultrasound [30]. But the relationship between PRDM9 and CAD were not clear. And, olfactory receptor family 14 subfamily a member 16 (DR14A16) was not reported in published articles. Therefore, PRDM9 and OR14A16 were need to be verified by more clinical examples. Meanwhile, *FUCA2, GRM1, SASH1, DMRTA1, IFT74*, and *TEK* were site in chromosome 9p21 and 6p24 regions, which were strongly associated with CAD. Among these, GRM1 and SASH1 have been found be associated with cardiovascular while others were firstly identified as related to CAD [31, 32]. According to the pLI and RVIS, the SBF1 and ZFHX3 with higher pLI and lower RVIS were considered as highly intolerant genes in the high-frequency variant genes. ZFHX3 were associated with atrial fibrillation and ischemic stroke [33]. Sun et al. found the rare genotypes only in CAD patients [34]. While, the relationship between SBF1 and coronary heart disease need further validation.

To investigate the possible mechanism of CAD-FL to CAD-DLL, KEGG pathway analysis was performed for the 46 high-frequency variant genes. The final result showed that lysosome may be associated with CAD-DLL. Lysosomes as organelles that break down biological macromolecules can mediate selective autophagy. The dysfunction of selective autophagy is associated with tumorigenesis, neurodegenerative diseases, metabolic disorders, etc [35]. . . Chaperone-mediated autophagy (CMA), a selective type of lysosomal degradation for intracellular proteins, could protect against atherosclerosis [36]. By measuring CMA activity in vivo, Madrigal-Matutea et al. found that CMA activity in plaque macrophages and smooth muscle cells gradually declines with plaque expansion. The knockout of lysosomal-associated membrane protein 2 A (LAMP2A) considerably aggravated the progression of atherosclerosis in mice fed a high-fat diet [36]. Therefore, we considered that high-frequency variant genes aggravated the development of CAD through functioning in the lysosome-related pathway.

According to the combined analysis, eight genes showed synchronized changes in DNA and protein levels. Among these, mannosidase alpha class 2a member 2 (*MAN2A2*) was overexpressed in patients with CAD-DLL as compared to that in controls with CAD-FL. Four patients with CAD-DLL carried the MAN2A2 variant, while none of the subjects with CAD-FL carried this variant. Recently, MAN2A2 has been identified as a factor associated with myocardial infarction and is upregulated during myocardial infarction [37, 38]; this finding is similar to our results. The expression levels of *MAN2A2* were associated with increased drug sensitivity according to the Genomics of Drug Sensitivity in Cancer (GDSC) database [39]. Cancer and cardiovascular diseases are risk factors for each other [40]. Therefore, MAN2A2 may be the potential pathogenic mechanism and an effective drug target for CAD-DLL. Both collagen 1A1 (COL1A1) and fibrillin-1 (FBN1) are associated with connective tissue diseases, and the change in their expression aggravates vessel damage [41, 42]. FBN1 variant (C1039G (+/-)) can also promote atherogenesis and a highly unstable plaque phenotype in apolipoprotein E-deficient (ApoE (-/-)) mice model of atherosclerosis [43]. Peptidylglycine α-amidating monooxygenase (PAM) is required for atrial secretory granule formation [44]. Although no studies have confirmed a direct relationship between PAM and CAD, low PAM expression may impair myocardial recovery after ischemia by dysfunction of atrial secretory granule formation. The remaining four genes may still be part of an important mechanism of CAD-DLL because of their alterations at the protein and DNA levels.

There were some limitations in this study. Firstly, this is a small example and observational study. Small sporadic example maybe results in missing important variant genes and is hard to obtain genes statistically associated with CAD-DLL. It is necessary to expand the clinical sample and focus on genetic variation in special populations. Secondly, this study lacks familial variation lineages. However, familial blood examples are difficult to obtain for some reasons, such as disease and death. Last but not least, the final results are not verified by experiment. We are planning to establish gene re-knockout in ApoE (-/-) mice to verify whether double knockout mice can obtain more severe coronary heart disease models.

## Conclusions

In conclusion, our study identified the important roles of multiple genes, including *PCLO* and *CCDC177* in CAD-DLL through bioinformatics screening and analysis. Combined DNA-protein analysis identified eight genes whose expression was altered at both protein and DNA levels that may be involved in CAD-DLL development. The lysosome-related pathways may play an important role in aggravating CAD-FL to CAD-DLL. Our study has great significance for understanding the mechanism of CAD-DLL disease. We hope that this study serves as a guiding tool for the diagnosis and prevention of CAD-DLL.

### Electronic supplementary material

Below is the link to the electronic supplementary material.

Supplementary Material 1

Supplementary Material 2

Supplementary Material 3

Supplementary Material 4

## Data Availability

The raw data have been uploaded to NCBI and the number of SRA file is SUB12209852.
