# Supplementary material for: Whole exome sequencing and proteomics-based investigation of the pathogenesis of coronary artery disease with diffuse long lesion
Source: J Cardiothorac Surg. 2024 May 7;19:280. doi: 10.1186/s13019-024-02760-5 (PMC11075290; doi:10.1186/s13019-024-02760-5)
Supplement: Supplementary file 2 — Supplementary Material 2 [file 13019_2024_2760_MOESM2_ESM.docx]

Table S1. The shared variants in the chromosome 9p21 and 6p24 regions.

| Gene name | Gene annotation | Site variant frequency | Homozygous/ Heterozygous variants | ACMG | HGVS | Variant site | ConsDetail | Nucleotide alteration |
| --- | --- | --- | --- | --- | --- | --- | --- | --- |
| FUCA2 | FUCOSIDASE, ALPHA-L, 2 | 1/20 | 0/1 | PM2 | NM_032020 c.G1121A (p.R374Q) | 6q24.2 exon5 | missense | C>T |
| FUCA2 |  | 1/20 | 0/1 | PM2 | NM_032020 c.A268G (p.M90V) | 6q24.2 exon2 | missense | T>C |
| GRM1 | GLUTAMATE RECEPTOR, METABOTROPIC, 1 | 1/20 | 0/1 | PM2 | / | 6q24.3 | . | GTTTCTC>G |
| GRM1 |  | 1/20 | 0/1 | PM2 | NM_001278064 c.C1767A (p.S589R)  NM_001278066 c.C1767A (p.S589R)  NM_001278067 c.C1767A (p.S589R)  NM_001278065 c.C1767A (p.S589R) | 6q24.3 exon7/8 | missense | C>A |
| GRM1 |  | 1/20 | 0/1 | PM2 | NM_001278064 c.A3386T (p.E1129V) | 6q24.3 exon8 | missense | A>T |
| SASH1 | STERILE ALPHA MOTIFS- AND SH3 DOMAIN-CONTAINING PROTEIN 1 | 1/20 | 0/1 | PM2 | NM_001346507 c.C437T (p.S146F)  NM_001346508 c.C437T:p.S146F,  NM_001346509 c.C314T (p.S105F)  NM_001346505 c.C1019T (p.S340F)  NM_015278 c.C1154T (p.S385F)  NM_001346506 c.C782T (p.S261F) | 6q24.3 exon3/10/11 | missense | C>T |
| SASH1 |  | 1/20 | 0/1 | PM2 | NM_001346508 c.G533A (p.R178H)  NM_001346509 c.G410A (p.R137H) | 6q24.3 exon3 | missense | G>A |
| DMRTA1 | DOUBLESEX- AND MAB3-RELATED TRANSCRIPTION FACTOR A1 | 1/20 | 0/1 | PM2 | NM_022160 c.C281T (p.T94M) | 9p21.3 exon1 | missense | C>T |
| DMRTA1 |  | 1/20 | 0/1 | PM2 | NM_022160 c.G1282A (p.V428I) | 9p21.3 exon2 | missense | G>A |
| DMRTA1 |  | 1/20 | 0/1 | PM2 | NM_022160 c.C1511T (p.P504L) | 9p21.3 exon2 | missense | C>T |
| IFT74 | INTRAFLAGELLAR TRANSPORT 74 | 1/20 | 0/1 | PM2 | NM_001099222 c.C535G (p.Q179E)  NM_001099223 c.C535G (p.Q179E)  NM_001099224 c.C535G (p.Q179E)  NM_001349928 c.C535G (p.Q179E)  NM_025103 c.C535G (p.Q179E) | 9p21.2 exon8 | missense | C>G |
| IFT74 |  | 1/20 | 0/1 | PM4 | NM_001099222 c.1524_1526del (p.508_509de)  NM_001099223 c.1524_1526del (p.508_509de)  NM_001349928 c.1524_1526del (p.508_509del)  NM_025103 c.1524_1526del (p.508_509del) | 9p21.2 exon18 | nonframeshift deletion | TATC>T |
| TEK | TEK TYROSINE KINASE, ENDOTHELIAL | 1/20 | 0/1 | PM2 | NM_000459 c.G316A (p.V106I)  NM_001290077 c.G316A (p.V106I) | 9p21.2 exon2 | missense | G>A |
| TEK |  | 1/20 | 0/1 | PM2 | NM_001290078 c.G1787C (p.G596A)  NM_001290077 c.G2099C (p.G700A)  NM_000459 c.G2228C (p.G743A) | 9p21.2 | missense | G>C |
