# Supplementary material for: Whole exome sequencing and proteomics-based investigation of the pathogenesis of coronary artery disease with diffuse long lesion
Source: J Cardiothorac Surg. 2024 May 7;19:280. doi: 10.1186/s13019-024-02760-5 (PMC11075290; doi:10.1186/s13019-024-02760-5)
Supplement: Supplementary file 3 — Supplementary Material 3 [file 13019_2024_2760_MOESM3_ESM.docx]

Table S2. The pLI and RVIS for the 33 high-frequency variant genes.

| Gene | pLI | RVIS |
| --- | --- | --- |
| CDCP2 | 0 | -0.13 (43.98%) |
| HSPBP1 | 0.06 | -0.18 (40.16%) |
| OR14A16 | NA | 0.64 (83.9%) |
| PCLO | 1 | -0.18 (40.57%) |
| PDE4DIP | 0 | 7.99 (99.95%) |
| PKD1 | 1 | NA |
| PKD1L3 | NA | NA |
| **SBF1** | 0.99 | -4.05 (0.17%) |
| WNK2 | 1 | NA |
| ASTN1 | 1 | -1.1 (6.98%) |
| CROCC | 0 | 2.43 (98.53%) |
| CTSH | 0 | 0.15 (64.61%) |
| FAM53A | 0 | NA |
| JMJD1C | 1 | -0.55 (19.81%) |
| MAN2A2 | 0 | -1.45 (3.93%) |
| MUC22 | 0.01 | NA |
| MYH14 | 0.04 | -2.24 (1.31%) |
| MYO7A | 0 | -2.51 (0.93%) |
| MYOM1 | 0 | 0.56 (81.68%) |
| MYOM3 | 0 | 1.16 (92.65%) |
| NDUFAF5 | 0 | -0.8 (12.33%) |
| NFATC1 | 0.16 | -1.85 (2.05%) |
| PEG3 | 0 | 1.51 (95.45%) |
| PIK3C2B | 1 | 0.45 (77.93%) |
| RTTN | 0 | 0.19 (66.28%) |
| SOX30 | 1 | 1.29 (93.85%) |
| SPHKAP | 0.94 | -0.8 (12.34%) |
| TENM4 | 1 | -1.7 (2.59%) |
| THADA | 0 | 0.45 (77.98%) |
| USP6 | 0 | 0.42 (76.96%) |
| XIRP2 | 0 | 1.46 (95.19%) |
| ZFHX4 | 1 | -1.74 (2.44%) |
| ZNF318 | 0.37 | -1.69 (2.62%) |
| ABCC11 | 0 | 1.62 (96.04%) |
| CCDC177 | NA | NA |
| GALC | 0 | 0.85 (88.48%) |
| LFNG | 0.1 | -0.05 (50.34%) |
| LPIN3 | 0 | 0.56 (81.72%) |
| MMP8 | 0 | 1.58 (95.75%) |
| MSH2 | 0.9 | -2.03 (1.68%) |
| MUC13 | 0 | NA |
| MYBBP1A | 0 | 2.3 (98.33%) |
| PRDM9 | 0 | 1.32 (94.07%) |
| TECPR2 | 0.58 | -0.47 (22.84%) |
| ZC2HC1C | 0 | 0.4 (76.31%) |
| **ZFHX3** | 1 | -4.26 (0.12%) |

pLI, loss-of-function intolerance; RVIS, Residual Variation Intolerance Score.
