# Supplementary material for: Whole exome sequencing and proteomics-based investigation of the pathogenesis of coronary artery disease with diffuse long lesion
Source: J Cardiothorac Surg. 2024 May 7;19:280. doi: 10.1186/s13019-024-02760-5 (PMC11075290; doi:10.1186/s13019-024-02760-5)
Supplement: Supplementary file 4 — Supplementary Material 4 [file 13019_2024_2760_MOESM4_ESM.docx]

Table S1 A list of the eight genes that vary in level of DNA and protein.

| Gene name | Gene annotation | Gene variant frequency | Site variant frequency | ACMG | HGVS | Variant site | ConsDetail | Nucleotide alteration |
| --- | --- | --- | --- | --- | --- | --- | --- | --- |
| PAM | Peptidylglycine alpha-amidating monooxygenase | 2/20 | 1/20 | PM2 | NM_000919 c.1157 A>G (p.D386G) | 5q21.1 exon13 | missense SNV | A>G |
|  |  |  | 1/20 | PM2, PP3 | NM_138822 c.2638 C>T (p.P880S) | 5q21.1 exon23 | missense SNV | C>T |
| CRISP3 | Cysteine-rich secretory protein 3 | 3/20 | 1/20 | PM1 | NM_001190986 c.568 T>C (p.Y190H) | 6p12.3 exon6 | missense SNV | A>G |
|  |  |  | 1/20 | PM1 | NM_001190986 c.452 G>C (p.G151A) | 6p12.3 exon5 | missense SNV | C>G |
|  |  |  | 1/20 | PM2, PP3 | - | 6p12.3 | - | T>G |
| C8G | Complement component 8, gamma subunit | 2/20 | 2/20 | - | NM_000606 c.12delT (p.P4fs) | 9q34.3 exon1 | frameshift deletion | CT>C |
| FBN1 | Fibrillin 1 | 2/20 | 1/20 | PM2, PP3 | NM_000138 c.7973 C>T (p.P2658L) | 15q21.1 exon64 | missense SNV | G>A |
|  |  |  | 1/20 | PM2 | NM_000138 c.3142 A>G (p.I1048V) | 15q21.1 exon26 | missense SNV | T>C |
| MAN2A2 | Mannosidase, alpha, class 2a, member 2 | 4/20 | 1/20 | PM1, PM2 | NM_006122 c.473 C>T (p.P158L) | 15q26.1 exon4 | missense SNV | C>T |
|  |  |  | 1/20 | PM1, PM2 | NM_006122 c.2014 C>T (p.R672C) | 15q26.1 exon14 | missense SNV | C>T |
|  |  |  | 1/20 | PM1, PM2, PP3 | NM_006122 c.2302 C>T (p.R768C) | 15q26.1 exon15 | missense SNV | C>T |
|  |  |  | 1/20 | PM1, PM2, PP3 | NM_006122 c.3115 C>T (p.R1039C) | 15q26.1 exon21 | missense SNV | C>T |
| COL1A1 | Collagen, type I, alpha 1 | 2/20 | 1/20 | PM2, PP3 | NM_000088 c.4039 G>A (p.D1347N) | 17q21.33 exon50 | missense SNV | C>T |
|  |  |  | 1/20 | PM2, PP3 | NM_000088 c.3536 C>T (p.P1179L) | 17q21.33 exon48 | missense SNV | G>A |
| MRC2 | Mannose receptor, c-type, 2 | 2/20 | 1/20 | PM1, PM2, PP3 | NM_006039 c.1843 C>T (p.R615C) | 17q23.2 exon12 | missense SNV | C>T |
|  |  |  | 1/20 | PM1, PM2 | NM_006039 c.3205 G>C (p.A1069P) | 17q23.2 exon22 | missense SNV | G>C |
| EMILIN3 | Elastin microfibril interfacer 3 | 2/20 | 1/20 | PM2, PP3 | NM_052846 c.1180 C>T (p.R394C) | 20q12 exon4 | missense SNV | G>A |
|  |  |  | 1/20 | PM2 | NM_052846 c.622 G>A (p.E208K) | 20q12 exon4 | missense SNV | C>T |

Abbreviation: ACMG, American College of Medical Genetics & Genomics; HGVS, Human Genome Variation Society.
