# Supplementary material for: Whole exome sequencing and proteomics-based investigation of the pathogenesis of coronary artery disease with diffuse long lesion
Source: J Cardiothorac Surg. 2024 May 7;19:280. doi: 10.1186/s13019-024-02760-5 (PMC11075290; doi:10.1186/s13019-024-02760-5)
Supplement: Supplementary file 1 — Supplementary Material 1 [file 13019_2024_2760_MOESM1_ESM.pdf]

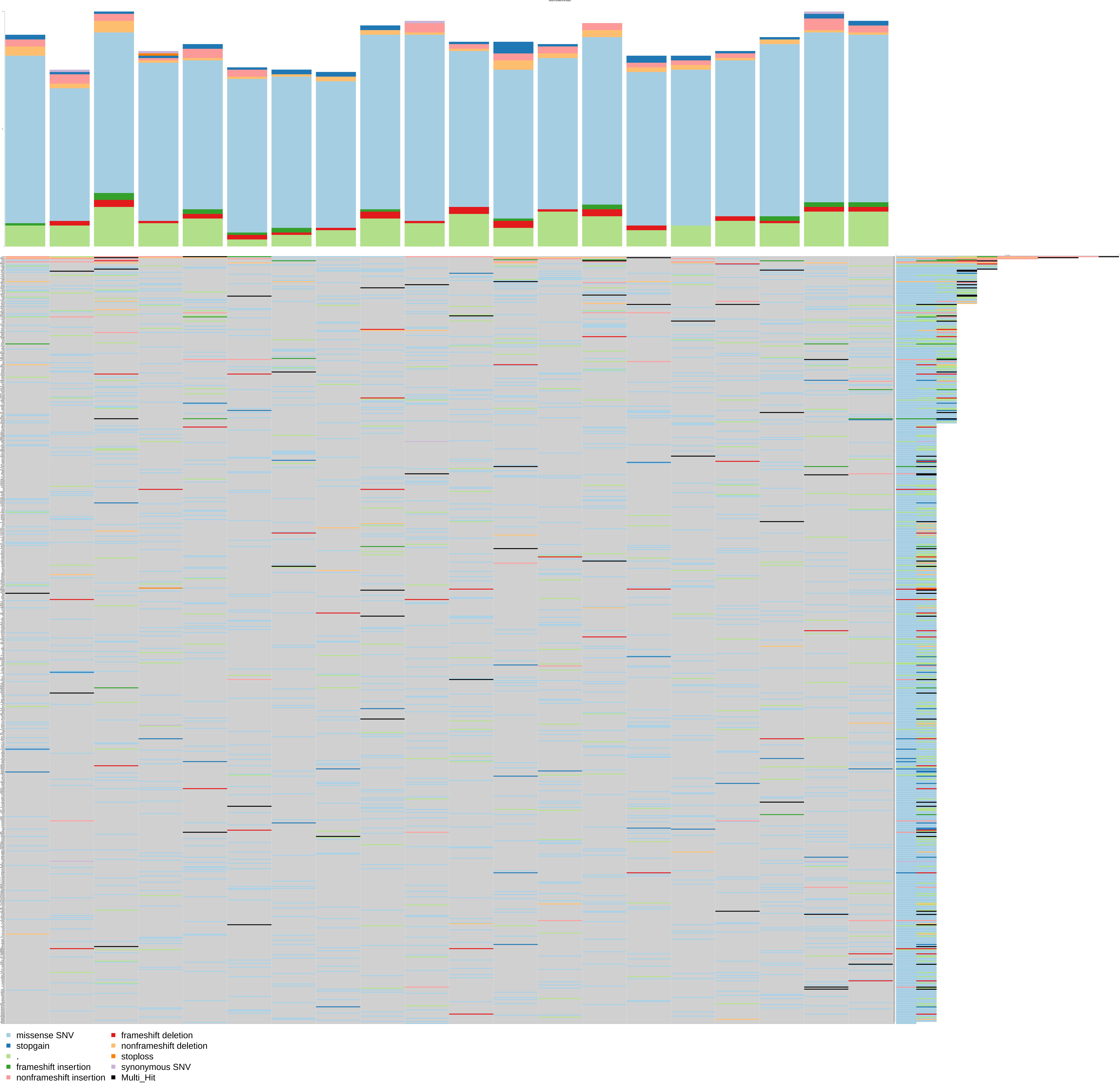

missense SNV
stopgain
.
frameshift insertion
nonframeshift insertion
frameshift deletion
nonframeshift deletion
stoploss
synonymous SNV
Multi_Hit
